# Relationships Between Anaerobic Performance, Field Tests and Game Performance of Sitting Volleyball Players

**DOI:** 10.1515/hukin-2015-0088

**Published:** 2015-01-12

**Authors:** Jolanta Marszalek, Bartosz Molik, Miguel Angel Gomez, Kęstutis Skučas, Judit Lencse-Mucha, Witold Rekowski, Vaida Pokvytyte, Izabela Rutkowska, Kalina Kaźmierska-Kowalewska

**Affiliations:** 1 The Jozef Pilsudski University of Physical Education in Warsaw, Faculty of Rehabilitation, Poland; 2 Technical University of Madrid, Spain; 3 Lithuanian Sports University in Kaunas, Lithuania

**Keywords:** disability sport, physical fitness

## Abstract

The aim of this study was to evaluate relationships between anaerobic performance, field tests, game performance and anthropometric variables of sitting volleyball players. Twenty elite Polish sitting volleyball players were tested using the 30 s Wingate Anaerobic Test for arm crank ergometer and participated in six physical field tests. Heights in position to block and to spike, as well as arm reach were measured. Players were observed during the game on the court in terms of effectiveness of the serve, block, attack, receive and defense. Pearson analysis and the Spearman’s rank correlation coefficient were used. The strongest correlations were found between the chest pass test and mean power and peak power (r=.846; p=.001 and r=.708; p=.0005, respectively), and also between the T-test and peak power (r= −.718; p=.001). Mean power correlated with the 3 m test (r= −.540; p=.014), the 5 m test (r= −.592; p=.006), and the T-test (r= −.582; p=.007). Peak power correlated with the 3 m test (r= −.632; p=.003), the 5 m test (r= −.613; p=.004), speed & agility (r= −.552; p=.012) and speed & endurance (r=−.546; p=.013). Significant correlations were observed between anthropometric parameters and anaerobic performance variables (p≤.001), and also between anthropometric parameters and field tests (p≤.05). Game performance and physical fitness of sitting volleyball players depended on their anthropometric variables: reach of arms, the position to block and to spike. The chest pass test could be used as a non-laboratory field test of anaerobic performance of sitting volleyball players.

## Introduction

Sitting volleyball is a Paralympic team game. The organization which is responsible for development of this sport as well as organizes world and continental championships is World ParaVolley. This game is created for people with disabilities such as amputation, Les Autres, poliomyelitis, focomelie, neuromuscular disease, multiple sclerosis, and people with minimal disability (MD). Sitting volleyball is a very fast and unpredictable game. The rules of this game are altered standing volleyball rules only in terms of a field (smaller −10 x 5 m) and a net (lower −1.15 m for males and 1.05 m for females). Moreover, players’ positions on the court are determined according to the positions their buttocks contact the ground (feet in standing volleyball) and one part of them should be on the court during the contact with the ball - attack, serve, block, receive and defense. Furthermore, athletes can block serves and are permitted to penetrate into the opponent’s space under the net during playing (World ParaVolley Official Sitting Volleyball Rules 2013–2016; Ng, 2012).

As well as in able-bodied sport, especially in team games, in sport for people with impairment, there is a need to seek relationships between factors, which influence sport results. Those factors could be anaerobic performance, physical fitness, tactics and technique of movement. For experts and coaches, it is important to use easy tests, which indicate the level of anaerobic performance or physical fitness and simultaneously could be applied on the field. In sitting volleyball there are not many studies in this area. Jadczak et al. (2010) evaluated the relationship between coordination abilities (time of simple reaction to visual stimulus - simple reaction, time of complex reaction to visual stimulus - complex reaction, effect of visual-motor coordination – the Piórkowski test, orientation ability - a cross matching test, attention divisibility, orientation ability – perception), general motor fitness (dynamic strength of upper limbs, static strength of hands, muscular endurance of upper limbs, muscular strength of body, body flexibility - back muscles, endurance-speed), special motor fitness (attack, serve, overhand pass, forearm pass, tip) and effectiveness of game (according to the formula proposed by Coleman (2002)) among sitting volleyball players. Those authors indicated that the following elements had strongest impact on effectiveness of sitting volleyball: body flexibility with endurance-speed (physical fitness), ball passes (overhand and forearm) and attack (special fitness), anticipation, orientation-perception and complex reaction (coordination abilities) (Jadczak et al., 2010). In another research, Jadczak et al. (2009) examined physical fitness (a flexed arm hang test, sit-ups in 30 s, a hand grip strength test, trunk lift, a medicine ball throw and an endurance and speed test were used) and coordination abilities among three groups of sitting volleyball players: able body people, players with medium and severe disabilities. Authors concluded that players with medium disability were the most appropriate athletes to play in this game (Jadczak et al., 2009). Molik et al. (2008) recommended three tests to evaluate physical fitness of sitting volleyball players: the 5 m sprint, the chest pass test, and the envelope (1.5 x 2.5 m) drill test. Unfortunately, none of this research considered the relationship between anaerobic performance, coordination abilities, physical fitness and game performance in sitting volleyball. Molik et al. (2008) only suggested that the new research should focus on physical fitness in context of coordination abilities and effectiveness of the game.

Presently, studies which indicate that physical fitness tests (field tests/non-laboratory tests) could be used to assess anaerobic performance (a laboratory test) cannot be found. It would be helpful for coaches to evaluate sports performance of sitting volleyball athletes using field tests, thus, there is a need to find non-laboratory tests which would assess short-efforts of sitting volleyball players. The aim of this study was to evaluate relationships between anaerobic performance, field tests, game performance and anthropometric variables of sitting volleyball players.

## Material and Methods

Twenty elite Polish sitting volleyball players were examined in this study: 12 males (35.5 ±7.22 y) and 8 females (30.5 ±11.38 y). They participated in an anaerobic performance test and physical fitness tests (field tests) during the team training camp before the World Championships in Sitting Volleyball in Elbląg in 2014. The 30 s Wingate Anaerobic Test on an arm crank ergometer (LODE ANGIO Groningen, Netherlands) with Software Package-Wingate v.1.07b (Groningen, Netherlands) was used to evaluate anaerobic performance of sitting volleyball players (Lode, 1998). The arm crank ergometer was set on a gymnastics ladder at a height in which the axis of rotation of the ergometer was horizontally aligned with the athlete’s shoulders. The testing protocol consisted of three parts: a 2 min warm up (60 rpm, 50 W), the main test i.e. 30 s of cranking at maximum speed, and 1 min of recovery on the ergometer (Lode, 1998). Anaerobic performance variables i.e. mean power output (MP), peak power output (PP), relative mean power (rMP), relative peak power (rPP) and the fatigue index (FI) were analyzed.

**Figure 1:**
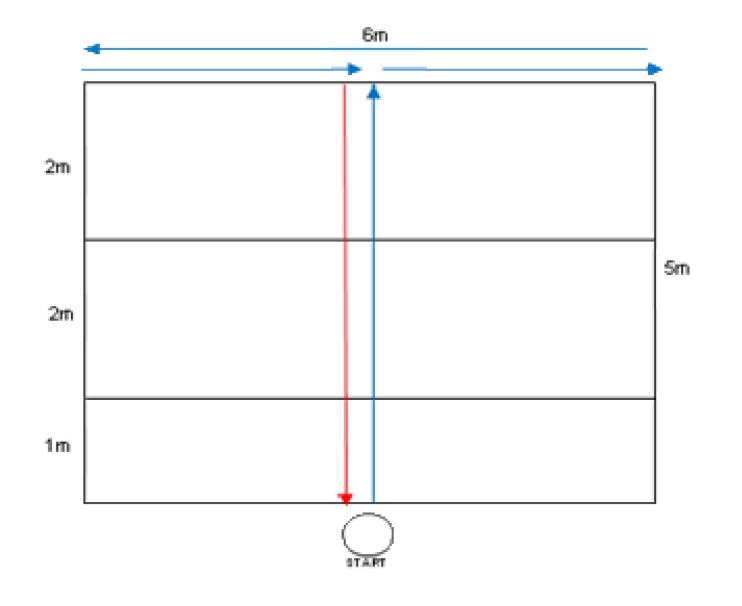
T-test (modification of Sassi et al., 2009)

**Figure 2:**
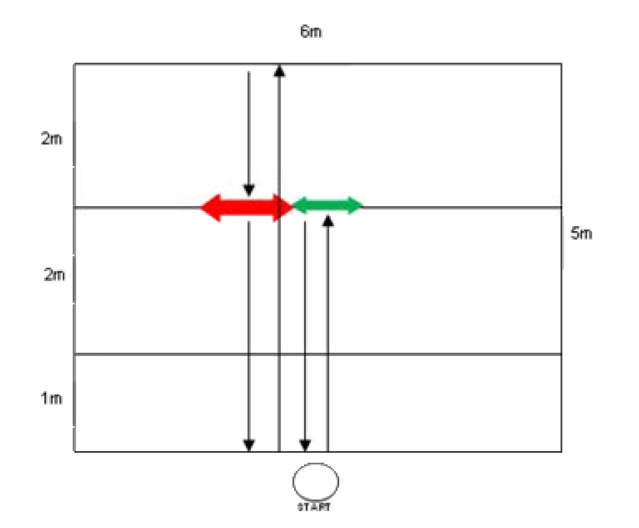
Speed & agility test (own concept)

**Figure 3:**
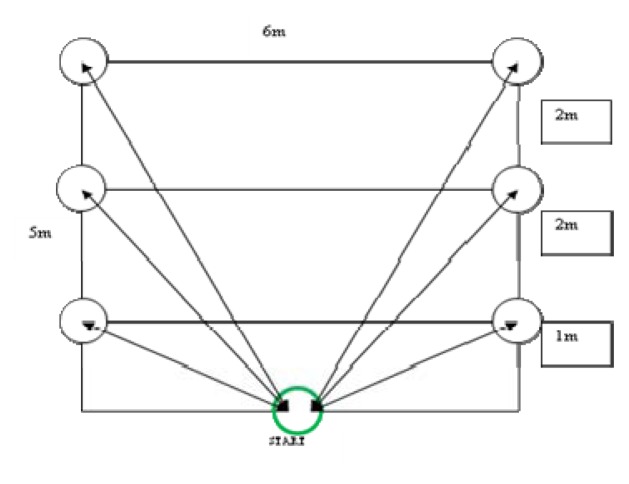
Speed & endurance test

The same group of athletes participated in six field tests: 3 and 5 m sprint tests, a chest pass test, a T-test (Figure 1; modification of Sassi et al., 2009), a speed & agility test (Figure 2; own concept), and a speed & endurance test (Figure 3). Additionally, anthropometric variables of athletes were measured: height in the position to block and spike, and arm reach. All three measurements were performed using a measuring tape. Height in the position to block (two hands elevated to the top) was measured in a sitting position facing a gate pole, from the ground to the highest part of hands. Height in the position to spike (one hand elevated) was measured in the same set, from the ground to the highest part of a hand. This set was the most comfortable for players and similar to the position during the game, as players can keep their lower limbs on the opponent’s field (below the net). Reach of arms was measured form the third finger of one hand to the longest part of the second hand and a player was set back to the wall with upper limbs raised laterally. All those three measurements were conducted three times with the accuracy of 0.5 cm and an average of each of those measurements was a recorded.

The next part of this study concerned the game performance. All matches, in which the Polish national team played, were recorded during the World Championships in Sitting Volleyball in Elbląg in 2014. Athletes were observed during the game on the court in terms of effectiveness of the attack (A_ef ), block (B_ef), block of serve (BS_ef), serve (S_ef), overhead receive (oR_ef), forearm receive (fR_ef), other receive (O_Ref), receive (R_ef), overhead defense (oD_ef), forearm defense (fD_ef), other defense (OD_ef), defense (D_ef). A libero player was excluded from the study due to the rules of sitting volleyball (no possibility to attack the ball from the first line). All observations were made by a professional league sport statistician and on the basis of the Games Observation Sheet in Sitting Volleyball. The obtained data were entered in the computer program DataVolley 2.0. Six formulas were used to count the effectiveness of the serve, block, attack, receive and defense (Coleman, 2002).

In this study all statistic analyses were performed with the use of IBM SPSS Statistics 21. Pearson analysis was used to find relationships between anaerobic performance parameters, field tests and anthropometric variables. The Spearman’s rank correlation coefficient was used to find relationships between game effectiveness and performance parameters, and also between field tests and anthropometric variables.

## Results

All significant correlations between anaerobic performance parameters and field tests as well as anaerobic performance parameters and anthropometric variables are shown in Table 1. Significant relationships were observed between field tests and anaerobic performance parameters (MP, PP; p≤.02). The strongest correlations were found between the chest pass test and mean power (MP), as well as peak power (PP; r=.846; p=.001 and r=.708; p=.0005, respectively), and also between the T-test and PP (r= −.718; p=.001). Mean power (MP) correlated with the 3 m sprint test (r= −.540; p=.014), the 5 m sprint test (r= −.592; p=.006) and the T-test (r= −.582; p=.007). Peak power (PP) correlated with the 3 m sprint test (r= −.632; p=.003), the 5 m sprint test (r= −.613; p=.004), the speed & agility test (r= −.552; p=.012) and the speed & endurance test (r=−.546; p=.013). Moreover, significant relationships were observed between anthropometric variables (range of reach, the position to block and to spike) and anaerobic performance parameters (MP, PP) (p≤.001) (Table 1).

Significant correlations were also observed between MP and the position to block (r=.723; p=.0003), the position to spike (r=.767; p=.0001) and range of reach (r=.732; p=.0002). Peak power (PP) also correlated with the position to block (r=.688; p=.001), the position to spike (r=.719; p=.0004), range of reach (r=.709; p=.0005). Significant correlations between anthropometric variables and field tests are shown in Table 2. The strongest relationships of all anthropometric variables (range of reach, the position to block and to spike) were found between the chest pass test (r=.725, r=.711, r=.738, p≤.0004, respectively) and the speed & agility test (r= −.530, r= −.505, r= −.532; p≤.004, respectively).

All significant correlations between anaerobic performance parameters, field tests, anthropometric variables and effectiveness are presented in Table 3. The strongest correlation between MP and effectiveness was observed in oR_ef, R_ef (r=.705, r=.771; p≤.005, respectively). Furthermore, the relationships were noticed between effectiveness of receiving the ball and all anthropometric variables (p≤.002) - the position to block, the position to spike and range of reach were correlated very strongly with oR_ef (r=.745, r=.707, r=.748; p≤.005, respectively) and with R_ef (r=.781, r=.767, r=.745; p≤.002, respectively).

Also field tests correlated with game effectiveness, i.e. the chest pass test correlated with oR_ef (r=.810; p=.0004), OR_ef (r=.810; p=.008) and R_ef (r=.856; p=.0001).

## Discussion

The aim of the present study was to evaluate the relationships between anaerobic performance, field tests, game performance and anthropometric variables of sitting volleyball players. Relationships between peak power output [W] and all field tests, as well as mean power output [W], 3 m and 5 m sprints [s], the chest pass test [m] and the T-test [s] confirmed that those selected field tests could be a tool for coaches to evaluate anaerobic performance among athletes in sitting volleyball in a non-laboratory setting. However, the strongest correlation was observed between the chest pass test, mean power output and peak power output, which means that this test could be the most appropriate tool for coaches to use. No other research on this issue in sitting volleyball has been found. In other sport disciplines for people with disability, for example in wheelchair basketball, analysis of anaerobic performance has been done, however, also in the context of the classification system (Molik et al., 2010). Molik et al. (2010) observed differences of anaerobic performance, which depended on the level of classification (the type of disability). In another study, Molik et al. (2013) tried to find a connection between anaerobic performance and selected field tests. The authors performed some analysis to determine the correlation between anaerobic performance and selected field tests and the classification system of athletes who played wheelchair basketball. Similarly as in the present study, the chest pass test correlated the strongest with mean power output and peak power output, which means that it could be a useful tool to indirectly assess anaerobic performance in wheelchair basketball players (Molik et al., 2013). In wheelchair rugby, Morgulec-Adamowicz et al. (2011) examined aerobic, anaerobic and skill performance in the context of the classification system, however, in conclusion they indicated that all those parameters were not dependent on the level of the classification system (the type of disability).

In the current study, a relationship between field tests and game performance was found. Passing the ball from the chest correlated with effectiveness of receives (the overhead receive, the forearm receive, other receive, all receives) and effectiveness of defense. It means that this test could be a good assessment tool providing that a player will be good at receiving the ball and at defense in the game. The same effectiveness parameters (without the forearm receive) correlated with mean power and all anthropometric variables: the position to block, the position to spike and range of reach. It is in line with the Strohkendl’s results (2001) indicating that physical potential influences sport performance. With regard to sitting volleyball effectiveness, one study has been found, however, the authors focused on the relationship between sports performance (service, reception, set, attack, block, and defense) and the classification system (people with disability, with minimal disability or able-bodied people) (Morres et al., 2006). Morres et al. (2006) observed that the level of sports performance did not decrease depending on the level of ability of athletes. Considering analysis of sitting volleyball in terms of game efficiency, other studies may be found (Vute, 1999; Häyrinen and Blomqvist, 2006; Häyrinen et al., 2010). Vute (1999) used Statistical Match Analysis (SMA) to evaluate sports performance, however, the author concentrated only on SMA and did not examine any other factors influencing the game. Similarly, Häyrinen and Blomqvist (2006) and Häyrinen et al. (2010) performed match analysis of elite sitting volleyball and female sitting volleyball at the international level, respectively, but they also concentrated only on the game variables. However, in other disciplines such as ice sledge hockey or wheelchair rugby, game performance could prove lack of necessity to divide athletes into groups in terms of the level/type of disability (Molik et al., 2012; Morgulec-Adamowicz et al., 2010).

In order to perform a holistic assessment of sitting volleyball players, a coach needs also to evaluate the technique of movement - attack, block, serve, receive, defense. Lack of those analyses in the present study is one of its limitations. Consequently, future research should focus on validity and reliability of technique tests - field tests to assess appropriate movement.

On the other hand, the chest pass test, which turned out the most suitable field tool for coaches to assess anaerobic performance and effectiveness of receives and defense of players, is not useful to assess athletes with hand impairment. It means that future studies should develop some new tests or retest those from present study (3 m and 5 m sprints [s], and the T-test [s]) to evaluate athletes with hand dysfunction.

## Conclusion

In conclusion, game performance and physical fitness of sitting volleyball players depend on their anthropometric variables: reach of arms, the position to block and to spike. This fact could be helpful for coaches to create a strong team in terms of game effectiveness.

Furthermore, the chest pass test could be used as a non-laboratory field test of anaerobic performance of sitting volleyball players, as it indirectly indicates effectiveness of the receive and defense.

There is a need to continue the research on a larger group of sitting volleyball athletes to confirm the present results.

## Figures and Tables

**Table 1:** Correlations between anaerobic performance and field tests as well as anaerobic performance and anthropometric variables

ANAEROBIC PARAMETERS	FIELD TESTS							ANTHROPOMETRIC VARIABLES		

		3m sprint [s]	5m sprint [s]	chest pass test [m]	T-test [s]	speed & agility test [s]	speed & endurance test [s]	BLOCK [cm]	SPIKE [cm]	Range of reach [cm]
**MP[W]**	r	−.540*	−.592**	.846***	−.582**	−.427	−.357	.723***	.767***	.732***
**PP [W]**	r	−.632**	−.613**	.708***	−.718***	−.552*	−.546*	.688**	.719***	.709***
**rMP [W/kg]**	r	−.195	−.255	.021	−.138	−.139	.075	.042	.071	.009
**rPP [W/kg]**	r	−.401	−.371	.118	−.422	−.369	−.249	.238	.246	.230
**FI [W/sec]**	r	−.339	−.319	.346	−.522*	−.398	−.774***	.557*	.544*	.559**

**MP** – mean power output; **PP** – peak power output; **rMP** – relative mean power; **rPP** – relative peak power; **FI** – fatigue index;

* p < 0.05

** p < 0.01

*** p < 0.001

**Table 2:** Correlations between field tests and anthropometric variables

ANTHROPOMETRIC VARIABLES	FIELD TESTS						

		3m sprint [s]	5m sprint [s]	chest pass test [m]	T-test [s]	speed & agility test [s]	speed & endurance test [s]
**BLOCK [cm]**	r	−.633**	−.687**	.711***	−.598**	−.505*	−.304
**SPIKE [cm]**	r	−.654**	−.732***	.738***	−.608**	−.532*	−.319
**Range of reach [cm]**	r	−.587**	−.632**	.725***	−.580**	−.530*	−.313

* p < 0.05

** p < 0.01

*** p < 0.001

**Table 3:** Correlations between anaerobic performance, anthropometric variables, field tests and effectiveness

		EFFECTIVENESS											

ANAEROBIC PERFORMANCE		A_ef	B_ef	BS_ef	S_ef	oR_ef	fR_ef	OR_ef	R_ef	oD_ef	fD_ef	OD_ef	D_ef
mean power [W]	r	.407	−.219	−.147	.225	.705**	.567	.740*	.771**	.524	.018	.149	.547*
peak power [W]	r	.481	.117	−.136	.126	.503	.567	.698*	.521	.345	.039	.226	.367
mean power/body mass [W/kg]	r	.228	.099	.348	−.086	−.134	.545	.254	−.036	.293	−.638*	.282	.007
peak power/body [W/kg]	r	.345	.242	.319	−.073	−.181	.519	−.038	−.166	.073	−.434	.360	−.144
rate to fatigue [W/sec]	r	.358	.258	−.563*	.115	.196	.500	−.017	.191	.117	.212	−.110	−.037
**ANTHROPOMETRIC VARIABLES**													
BLOCK [cm]	r	.359	−.029	.045	.188	.745**	.351	.788*	.781**	.454	−.012	.215	.587*
SPIKE [cm]	r	.233	−.135	−.085	.215	.707**	.395	.734*	.767**	.496	−.231	.137	.533*
Range of Reach [cm]	r	.329	−.071	.019	.145	.748**	.544	.822**	.745**	.334	.050	.378	.514
**FIELD TESTS**													
3m sprint [s]	r	−.376	.104	−.087	−.424	−385	−.317	−.286	−.363	−.205	.261	.058	−.182
5m sprint [s]	r	−.371	.086	.144	−.418	−.495	−.250	−.235	−.543*	−.407	.322	.097	−.385
chest pass test [m]	r	.339	−.144	.114	.070	.810**	.683*	.810**	.856**	.407	.173	.350	.673**
t-test [s]	r	−.451	.055	.179	−.380	−.451	−.183	−.227	−.446	−.332	.191	−.071	−.257
speed & agility test [s]	r	−.490	.227	.107	−.241	−.521	−.550	−.294	−.609*	−.521	.371	−.356	−.389
speed & endurance test [s]	r	−.336	.020	.552	−.539*	−.257	−.317	.218	−.187	.011	−.159	.291	.130

**A_ef** – effectiveness of attack; **B_ef** – effectiveness of block; **BS_ef** – effectiveness of block of serve; **S_ef** – effectiveness of block; **oR_ef** – effectiveness of overhead receive; **fR_ef**- effectiveness of forearm receive; **O_Ref** – effectiveness of other receive; **R_ef** – effectiveness of receive; **oD_ef** – effectiveness of overhead defense; **fD_ef** – effectiveness of forearm defense; **OD_ef** – effectiveness of other defense; **D_ef** – effectiveness of defense; **MP** – mean power output; **PP** – peak power output; **rMP** – relative mean power; **rPP** – relative peak power; **FI** – fatigue index;

* p < 0.05

** p < 0.01

*** p < 0.001
